# Carotico-Clinoid Foramen and Associated Clinical Significance: Comprehensive Review

**DOI:** 10.7759/cureus.12828

**Published:** 2021-01-20

**Authors:** Rajani Singh

**Affiliations:** 1 Anatomy, Uttar Pradesh University of Medical Sciences, Etawah, IND

**Keywords:** carotico-clinoid foramen, dural fold, anterior clinoid process, middle clinoid process

## Abstract

The carotico-carotid foramen occurs due to the ossification of the carotico-carotid ligament or dural fold stretching between the anterior and middle clinoid processes. The foramen is closely related to the cavernous sinus, pituitary gland, sphenoidal air sinus, and internal carotid artery irrigating the major part of the cerebrum (part of the brain). Due to the close association of the carotico-clinoid foramen with the aforementioned intracranial structures that may be affected by the formation of an anomalous foramen creating various neurological complications. Therefore, the study was carried out. The aim of the study is to consolidate all the data relating to the carotico-clinoid foramen to make it available to neurosurgeons as a ready reference.

For this, the literature was surveyed using various databases, and the terms related to the carotico-clinoid foramen and associated clinical significance have been elucidated. The literature survey brought out that this foramen is congenital in origin and it is of three types, of which the third type is the most dangerous, as it may cause severe hemorrhage, creating a plethora of complications. The other two types may compress the internal carotid artery, causing ischemic changes in the brain.

The information provided by this study will be of utmost use to neurosurgeons to carry out surgical interventions in the vicinity of the carotico-clinoid foramen.

## Introduction and background

The lesser wing of the sphenoid is a very crucial bone, as it is frequently used as a landmark in neurosurgery around the middle cranial fossa. The projecting medial ends of the lesser wing are known as the anterior clinoid process (ACP); they are connected to the middle clinoid process by the carotico-clinoid ligament or dural fold. Complete ossification of the ligament or dural fold culminates into the carotico-clinoid foramen (CCF) [1]. CCF is significant because it is situated in close proximity to the cavernous sinus, sphenoid sinus, pituitary gland [2], and intimately related to the internal carotid artery (ICA). The inter-clinoid ligament between the and posterior clinoid processes sometimes also ossifies, forming the inter-clinoid foramen [2]. These are examples of inconstant foramina in contrast to constant foramina (foramen rotundum, foramen ovale, foramen spinosum, and optic canal) present in the middle cranial fossa.

The ICA is an important source of irrigation to the major part of the cerebrum of the brain. The ICA that passes through the cavernous sinus follows its path along the medial to anterior clinoid process. Thus, the ICA is intimately related to the anterior clinoid process in addition to structures mentioned in the preceding paragraph. Therefore, neurosurgery involving the anterior clinoid process and thereby CCF is likely to damage the ICA and surrounding intracranial structures. Injury to ICA may disrupt regular arterial supply to the brain and injury to intracranial structures in close proximity of the CCF may create a plethora of neurological complications to the extent of fatality in many cases. The presence of complete or partial CCF may further compress or impinge the ICA causing degeneration or bleeding. Moreover, the cavernous sinus, sphenoid sinus, and pituitary gland situated in close proximity of the CCF may be affected, making radiological interpretation subjective and neurosurgical access and intervention difficult. This may enhance the chances of iatrogenic injuries.

Therefore, considering the immense serious clinical complications of ICA due to compression or impingement by bony spurs of a variant morphology of CCF, the study has been carried out to consolidate the anatomical variations associated with the CCF to facilitate neurosurgeons for ready reference.

## Review

Classification and incidence of carotico-clinoid foramen

**Figure 1:**
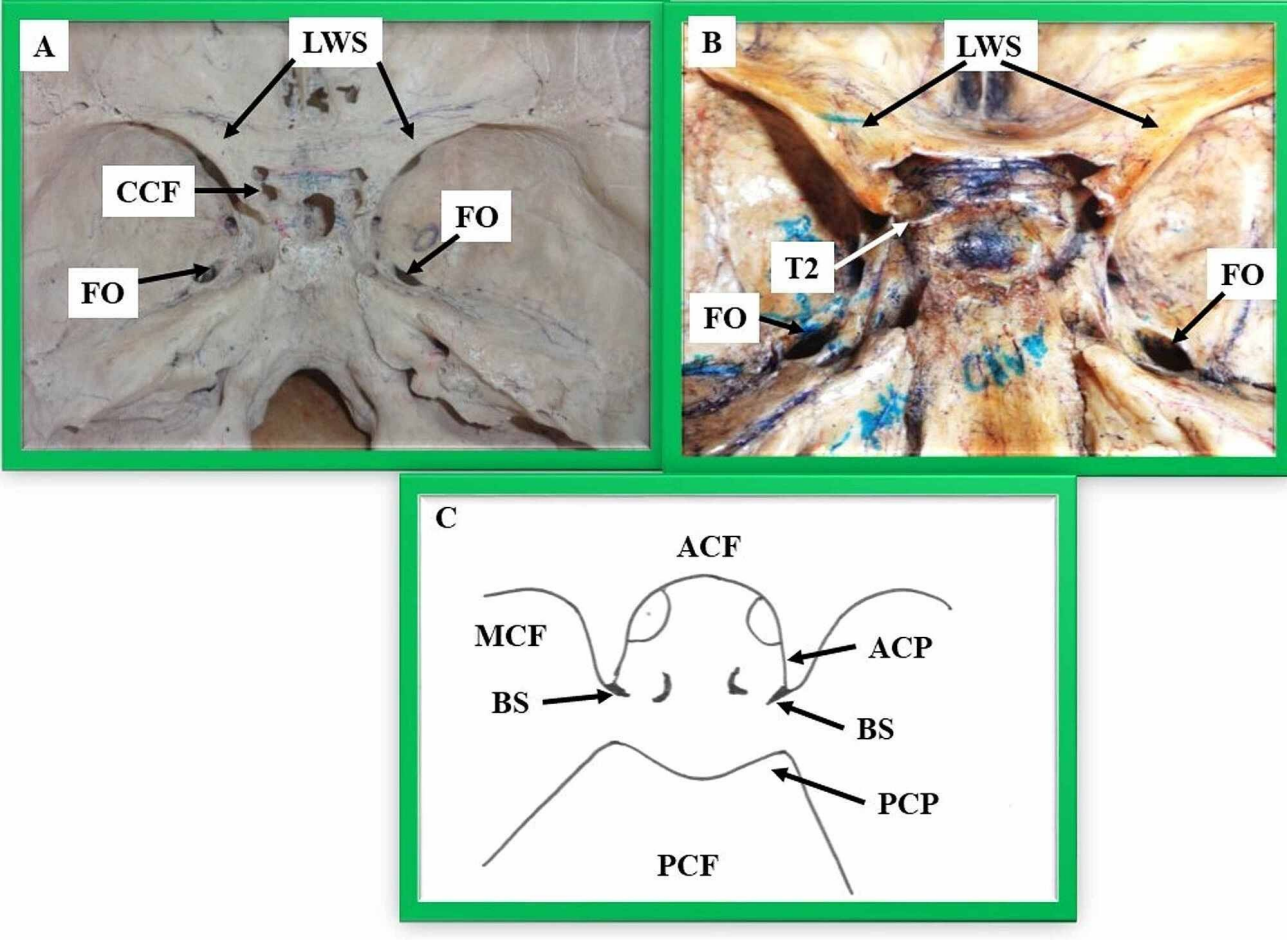
1A showing a type-I carotico-clinoid foramen, 1B showing a type-II carotico-clinoid foramen, and 1C showing a type-III carotico-clinoid foramen CCF - complete carotico-clinoid foramen, LWS - lesser wing of the sphenoid, FR - foramen rotundum, FO - foramen ovale, T 2 - type-II carotico-clinoid foramen, BS - bony spur projecting from the anterior and middle clinoid processes, ACP - anterior clinoid process, ACF - anterior cranial fossa, MCF - middle cranial fossa, PCF - posterior cranial fossa, PCP - posterior clinoid process

The carotico-clinoid ligament ossifies partially or fully to form a complete or incomplete foramen. Depending on the degree of ossification of the carotico-clinoid ligament/dural fold, Keyes classified these foramina into three categories: Type-I: Complete foramen (Figure 1A), Type-II: Contact type where foramen is present with a suture/break between the two ends (Figure 1B), and Type-III: Incomplete where a bony spur projects from the anterior and middle clinoid processes (Figure 1C) [3].

CCF has been extensively studied by various investigators. Ozdogmus et al. studied 50 autopsy bodies (100 sides) and observed a complete foramen on 27 sides and incomplete on 18 sides [2]. Erturk et al. observed the complete type in 4.09%, contact type in 4.68%, and incomplete type in 14.91% out of a total of 171 Turkish specimens [4]. Three more studies were conducted in the Turkish population [5-7], of which the highest incidence of complete foramina was 27%, detected by Gurun et al. [5] and the least incidence of 3% was observed by Cireli et al. [7]. A study in 270 Portuguese adult skulls revealed that the fusion between the anterior and middle clinoid processes (CCF) occurred in 17 cases (6.27%) and out of that, in 11 cases (4.05%), it was observed bilaterally [8]. These scientists did not give the incidence according to the classification but simply mentioned the sidewise occurrence.

A study revealed the complete carotico-clinoid canal in 4.1% and incomplete in 11.6% in 73 Korean skulls [9]. Freire et al. studied 80 Brazillian dry skulls and detected this foramen in 6.25% of specimens [10]. Plenty of literature describes the occurrence of this foramen in the Indian population [11-19]. The incidence of this foramen ranges from 6% to 37% in the Indian population. In South India, the incidence lies between 6% and 20%, and the incidence in the Gujrat region was found to be between 3% and 9%. The analysis exhibits that the incidence of CCF was observed to vary with ethnicity. Besides the variations observed in the populations of different countries, variations were also observed in the populations of different Indian states. This may be due to different sample sizes, personal observation errors in designating complete/incomplete foramen, and the genetic composition of the population.

Etiology of foramina

The ossification of ligaments is normally an age-related physiological process. The carotico-clinoid foramen and the interclinoid bony bridge were demonstrated in fetal and infant skulls, suggesting CCFs as the congenital anomalies [20-21] developing during intrauterine life. Thus, these entities are not related to age and may be caused by a mutation in certain genes. In addition, CCF is more commonly observed in persons with hormonal disturbance, the developmentally disabled, criminals, and epileptics [4].

Clinical implications of the carotico-clinoid foramen

The ICA is located medially to the anterior and middle clinoid process. Type-I and II may compress or stretch the ICA, causing irregular ischemic changes producing headaches and other neurological inconveniences resulting from inadequate blood supply to the brain [2,11,13]. A study by Das et al. revealed that CCF almost always alters the morphology of ICA [22], and transformations in the ICA may squeeze the cavernous sinus because of its close proximity to the cavernous sinus [23-24], producing severe neurological complications. In the case of a paraclinoid aneurysm, an anterior clinoidectomy is the treatment of choice [25-26], which becomes risky and challenging due to the presence of a bony CCF, as it may lead to heavy hemorrhage in the vicinity of the ACP [23]. After an anterior clinoidectomy, the clinoid space becomes exposed. This space is triangular in shape [24], and its size changes on the basis of the size of the anterior clinoid process and the ICA. The triangular area was located close to the cavernous sinus [27], and it was defined as intracavernous by various authors [25,28]. The anterior clinoid process is separated during surgical interventions involving the clinoid space, cavernous sinus [22], and other closely placed structures that may be affected. The existence of CCF may cause impediments to access ACP during neurosurgery [4]. Anterior clinoidectomy is carried out to access the cavernous sinus for treatment of a proximal ICA aneurysm. This clinoidectomy becomes difficult due to the presence of bony CCF. In addition to this, the clinoid part of the ICA and oculomotor nerve may be injured during the pruning of the ACP [2,11,13]. Occasionally, the ACP may be pneumatised or the bone may have a varying degree of density. So the ACP should be carefully axed so as to avoid injuring the ICA and optic nerve. Therefore, the ACP, ICA, and optic nerve should be examined radiologically preoperatively [4,11,13].

Type-III CCF is more dangerous, as spurs formed due to incomplete ossification may impinge on ICA during the pulsating of the ICA, causing severe bleeding.

## Conclusions

Various types of CCF may create a misinterpretation of radiographs if this anomaly is unknown. Thus, detailed and thorough awareness of various forms of CCF is essential to neurosurgeons to avoid injury to various structures during neurosurgical interventions and to radiologists to improve their interpretation of radiographs. The anterior clinoid process is usually removed in order to approach the cavernous sinus and related structures in neurosurgery. The presence of the CCF and interclinoid osseous bridge creates obstacles while approaching the anterior clinoid process in regional neurosurgery and increases the risks, especially if an aneurysm of the internal carotid artery is present. Further, a high incidence of these structures has been reported in subjects with hormonal disturbance, the developmentally disabled, criminals, and epileptics. Therefore, detailed anatomical knowledge of the carotico-clinoid foramen and interclinoid osseous bridge is of paramount importance to obtain successful results while operating on this region for various neurological conditions.
